# Supplementary material for: Single-cell and spatial RNA sequencing reveal the spatiotemporal trajectories of fruit senescence
Source: Nat Commun. 2024 Apr 10;15:3108. doi: 10.1038/s41467-024-47329-x (PMC11006883; doi:10.1038/s41467-024-47329-x)
Supplement: Supplementary file 1 — Supplementary Information [file 41467_2024_47329_MOESM1_ESM.pdf]

Supplementary Information

Single-Cell and Spatial RNA Sequencing Reveal the Spatiotemporal Trajectories of Fruit Senescence

Xin Li[1,2,3], Bairu Li[1], Shaobin Gu[1], Xinyue Pang[4], Patrick Mason[2], Jiangfeng Yuan[1], Jingyu Jia[1], Jiaju Sun[1], Chunyan Zhao[5,*], Robert Henry[2,*]

[1] College of Food and Bioengineering, Henan University of Science and Technology, Luoyang 471023, China.
[2] Queensland Alliance for Agriculture & Food Innovation, Queensland Biosciences Precinct, The University of Queensland, St Lucia QLD 4072, Australia.
[3] National Demonstration Center for Experimental Food Processing and Safety Education, Luoyang 471023, China.
[4] College of Medical Technology and Engineering, Henan University of Science and Technology, Luoyang 471023, China
[5] Institute of Environment and Health, Jianghan University, Wuhan 430056, China.

*Corresponding authors: Chunyan Zhao, 13025569@qq.com; Robert Henry, robert.henry@uq.edu.au

Inventory of Supplementary Information

Supplementary Information 1

Inventory of Supplementary Information 1

SUPPLEMENTARY FIGURES 2

Supplementary Figure 1: Optimization of spatial transcriptomics permeabilization conditions. 2

Supplementary Figure 2: Correlation analysis between spatial transcriptomics and single-cell transcriptomics. 4

Supplementary Figure 3: Subcluster analysis of exocarp and mesocarp cells. 6

Supplementary Figure 4: Senescent trajectories of exocarp cells and mesocarp cells. 8

Supplementary Figure 5: Metabonomic analyses of different components in the pericarp of H. undatus. 9

SUPPLEMENTARY TABLES 10

Supplementary Table 1. Summary of the cell data in *H. undatus* pericarp samples before and after filtering. 10

Supplementary Table 2. Cluster information for 13 cell clusters of *H. undatus* pericarp cells. 11

SUPPLEMENTARY FIGURES

Supplementary Figure 1

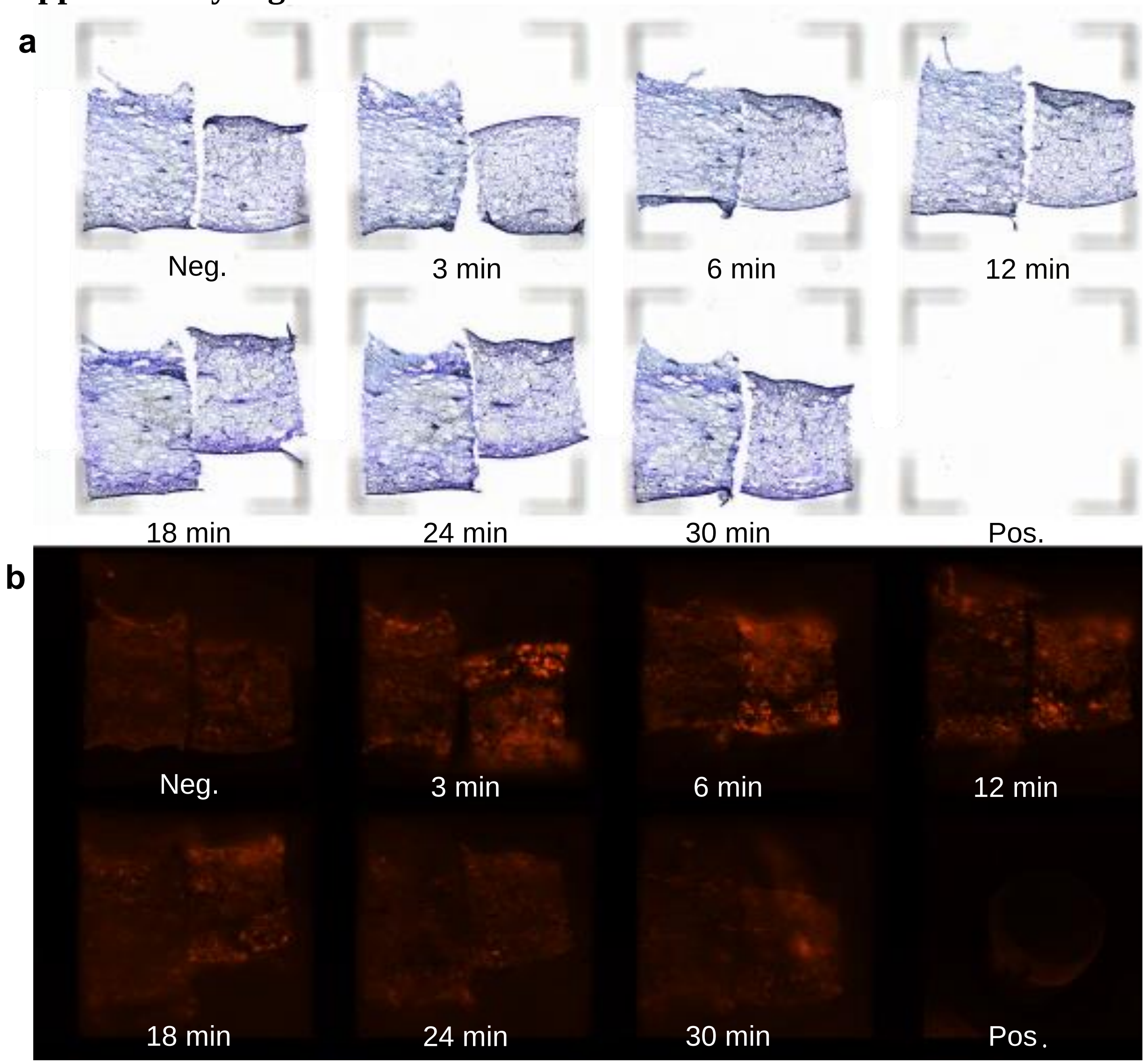

Supplementary Figure 1: Optimization of spatial transcriptomics permeabilization conditions. *Three independent experiments were repeated with similar results.*

a. Toluidine Blue staining. **b.** Fluorescence staining.

Supplementary Figure 2

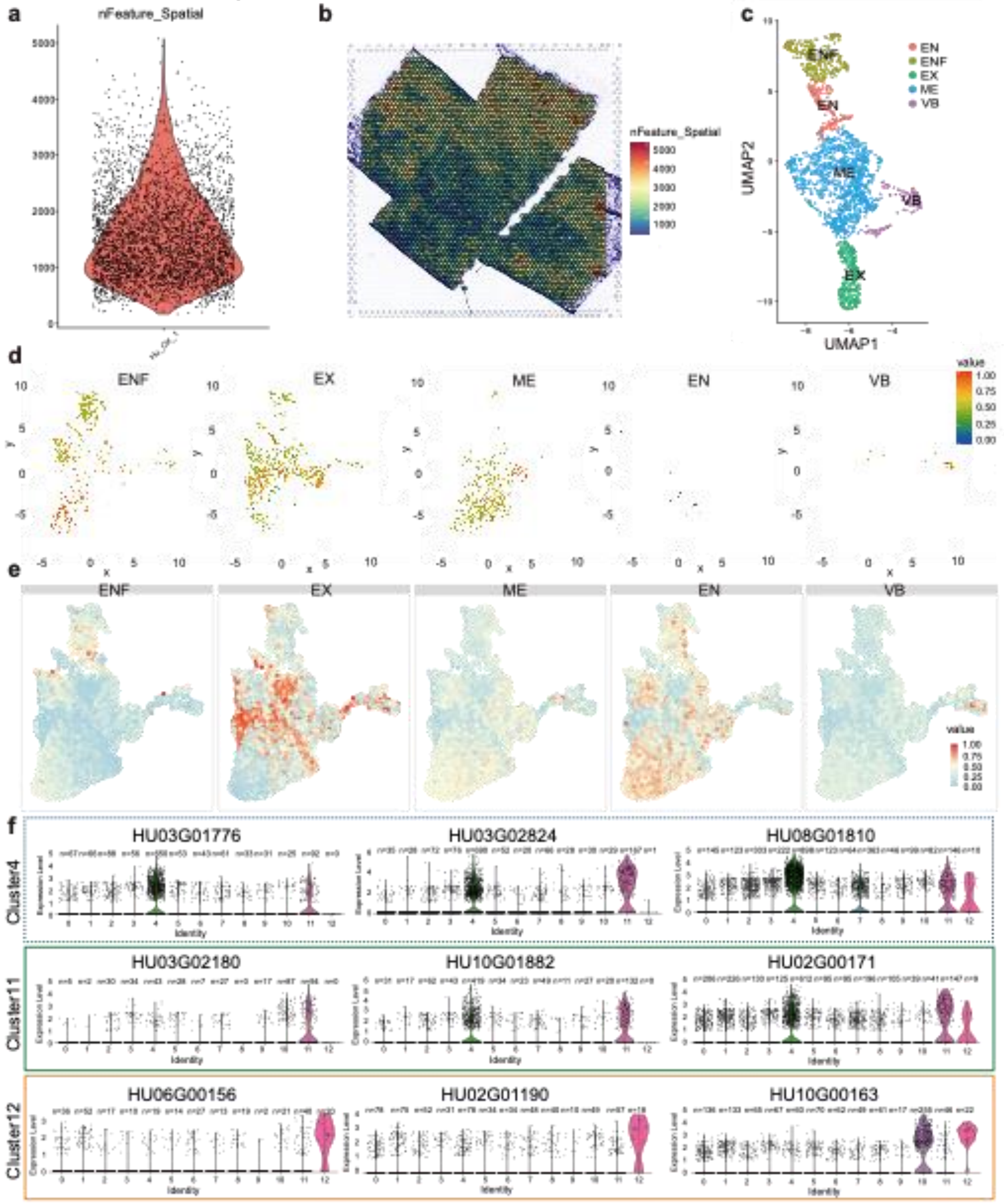

Supplementary Figure 2: *Correlation analysis between spatial transcriptomics and single-cell transcriptomics.*

a and b. Acquisition of effective genes in spatial transcriptomics (stRNA-seq). **c.** UMAP plot displaying various cell clusters in stRNA-seq. **d and e**. RCTD and CARD methods for correlation analysis between stRNA-seq and scRNA-seq. Heat dot plots illustrate EX, ME, and other five components, respectively. **f.** Violin plot displaying the expression of marker genes of clusters 4, 11, and 12 in each cluster. The violin plot showed six data nodes for each set of data, arranged from largest to smallest, namely the maximum value (upper edge), the upper quartile, the median, the lower quartile, and the minimum value (lower edge).

Supplementary Figure 3

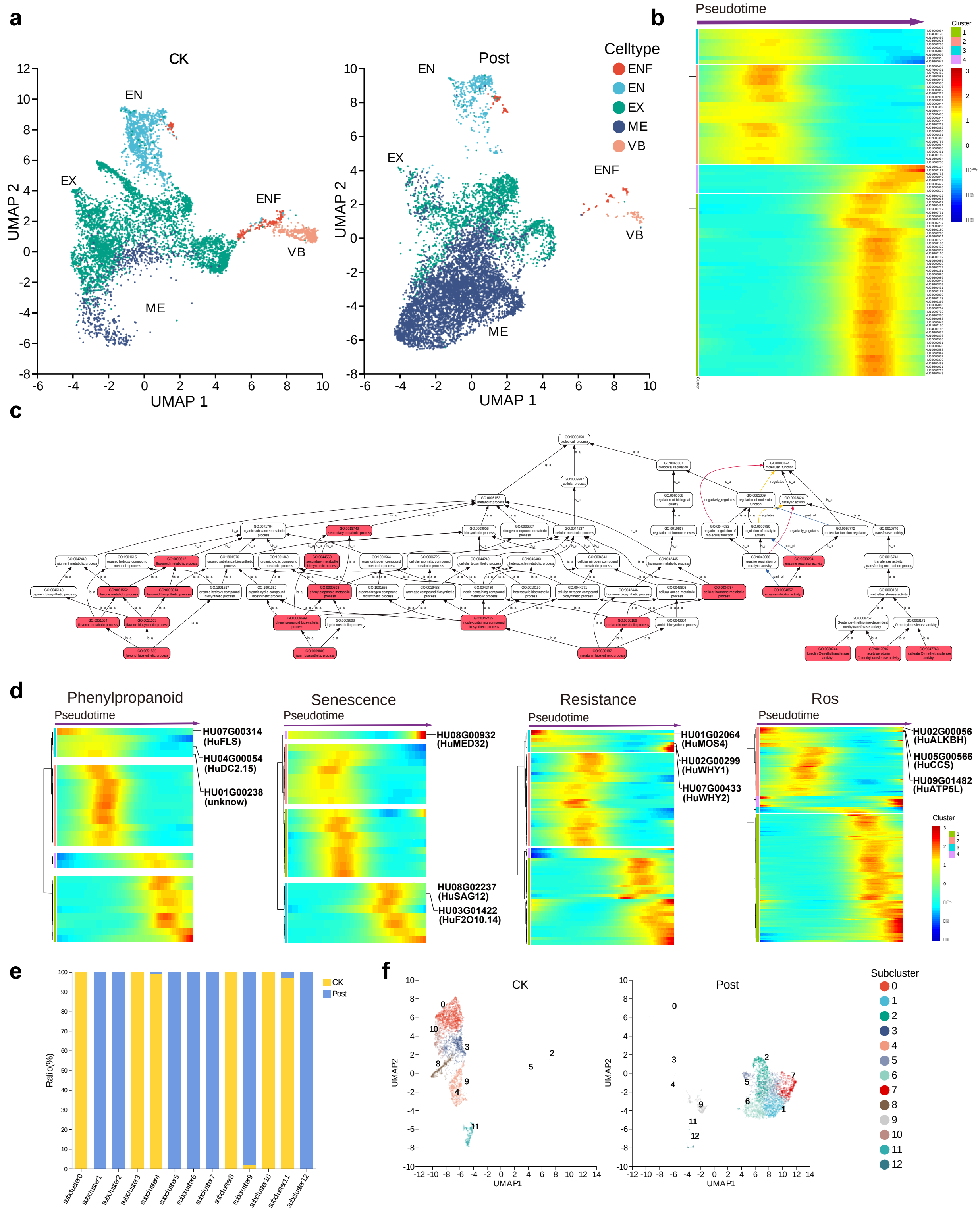

Supplementary Figure 3: Subcluster analysis of exocarp and mesocarp cells.

a. UMAP plots for CK and Post samples. **b.** Clustering of differentially expressed genes along a pseudotime progression of genes. **c.** DAG of GO terms in the cluster 2 of pseudotime related genes. The figure illustrates a subset of the molecular function and biological process DAG for flavonoids related methyltransferase activities (GO:0030744, GO:0017096, and GO:0047763), flavonol biosynthetic process (GO:0051555), and lignin biosynthetic process (GO:0009809). The ancestors of them were highlighted back to the root of the biological process (GO:0008150) and molecular function (GO:0003674) ontologies via arrows. **d.** Heatmap showing gene expression pattern of genes related to phenylpropanoids, senescence, resistance, and ROS during differentiation of pericarp cells along pseudotime. **e.** Subpopulation analysis of cells highly correlated with senescence and resistance in exocarp and mesocarp components. **f.** UMAP plots illustrating the 13 subclusters obtained in Supplementary Figure 3d.

Supplementary Figure 4

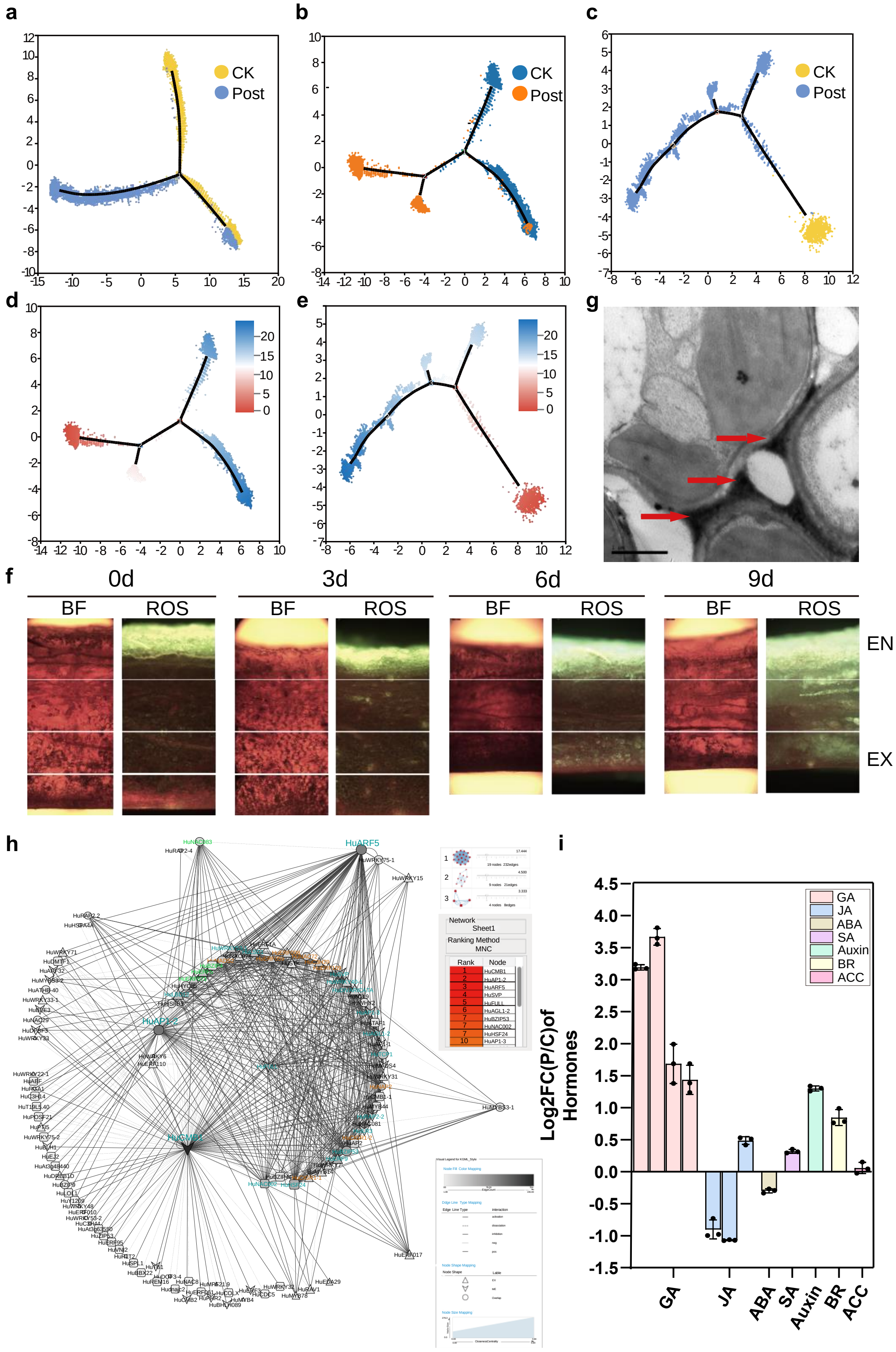

Supplementary Figure 4: Senescent trajectories of exocarp cells and mesocarp cells.

a. Distribution of CK and Post cells on the pseudotime trajectory of cells highly correlated with senescence. **b and c.** The pseudotime differentiation trajectory of EX cells and ME cells were visualized and displayed according to the samples, respectively. **d and e.** The pseudotime differentiation trajectory of EX cells and ME cells were visualized and displayed based on the differentiation time, respectively. Different colors of latent time represent different differentiation times, with darker shades of red indicating earlier times, and darker shades of blue indicating later times. **f.** Fluorescence changes in ROS localization observed under a fluorescence microscope with extended storage time, using DCF staining. **g.** Accumulation of H_2O_2 during pathogen infection-induced oxidative burst in plants. Scale bar: 1 μm. n= 3 independent experiments were repeated with similar results in supplementary figures 4f and g. **h.** The Gene Regulatory Network (GRN) is inferred from the dynamic expression of top 100 genes at pseudo-temporal branching points and 529 transcription factors integrated dynamically expressed across senescence differentiation pseudotime with a parameter cutoff of 2.0. Solid and dotted lines represent positive and negative regulation, respectively. Node size corresponds to the predicted connectivity. Node shapes and colors were consistent with Figure 5J. **i.** Mass spectrometry detection of hormone levels in senescent *H. undatus* samples. The GA molecules are Gibberellin A1, A3, A4 and A7 from left to right. The JA molecules are Methyl Jasmonate, Jasmonic acid, and Jasmonic Acid-Isoleucine from left to right. Three independent experiments were repeated with similar results. Data are presented as mean values ± SD.

Supplementary Figure 5

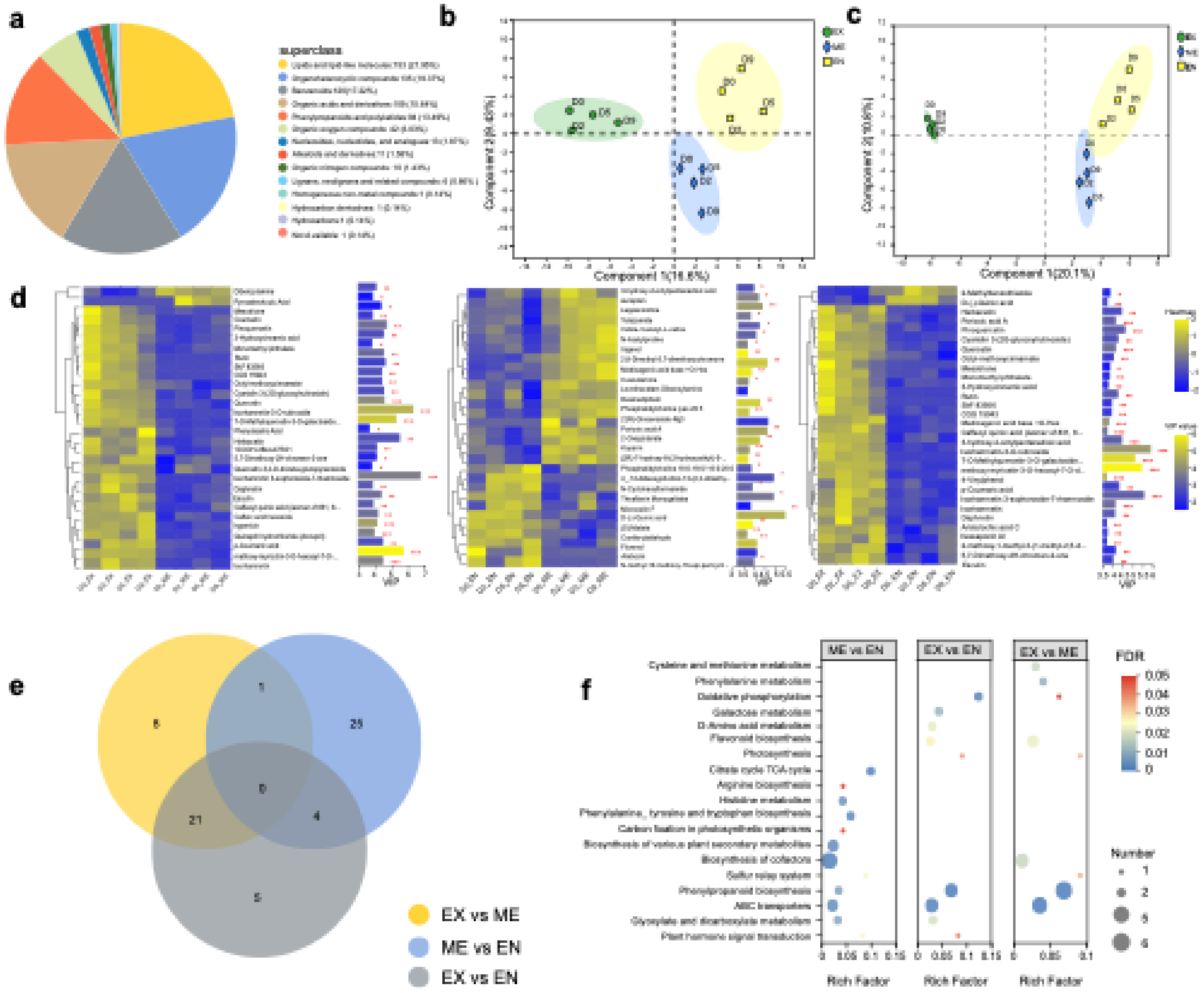

Supplementary Figure 5: Metabonomic analyses of different components in the pericarp of H. undatus.

a. Classification of metabolites identified in the pericarp by HMDB. **b and c.** PLS-DA analysis of metabolites identified in different samples under positive and negative ion modes, respectively. **d.** Expression and VIP values of top 30 differentially expressed metabolites among the three components (VIP ≥ 1.0), from left to right: Exocarp vs. Mesocarp, Mesocarp vs. Endocarp, Exocarp vs. Endocarp. “*” and “**” mean $p < 0.05$ and $p < 0.01$ in the t-test, respectively. **e.** Venn diagram of overlapping differentially expressed metabolites among the three components. Source data are provided as a Source Data file. **f.** KEGG enrichment analysis of differentially expressed metabolites among the three components. The p-values were calculated based on one-sided hypergeometric model and p-adjust method (BH) was used in the KEGG analysis.

SUPPLEMENTARY TABLES

Supplementary Table 1. Summary of the cell data in *H. undatus* pericarp samples before and after filtering.

Sample	CK	Post
nCount_RNA	4214	5322
nFeature_RNA	200-2089	200-2303
Percent_double_cell	6.35%	7.02%
Num_Orig	6738	9179
Num_filterd	5646	7670
Mean Reads per Cell	62,762	44,246
Median Genes per Cell	708	777
Valid Barcodes	91.60%	95.60%
Fraction Reads in Cells	49.10%	64.90%
Sequencing Saturation	60.60%	56.20%
Reads Mapped to Genome	95.40%	91.80%
Reads Mapped Confidently to Exonic Regions	30.90%	30.60%

Supplementary Table 2. Cluster information for 13 cell clusters of *H. undatus* pericarp cells.

Cluster	CK	Post	cluster_num	Ratio_cluster (%)
0	327(5.79%)	1625(21.19%)	1952	14.66%
1	34(0.60%)	1832(23.89%)	1866	14.01%
2	1134(20.09%)	505(6.58%)	1639	12.31%
3	858(15.20%)	444(5.79%)	1302	9.78%
4	881(15.60%)	363(4.73%)	1244	9.34%
5	530(9.39%)	666(8.68%)	1196	8.98%
6	248(4.39%)	833(10.86%)	1081	8.12%
7	721(12.77%)	342(4.46%)	1063	7.98%
8	5(0.09%)	884(11.53%)	889	6.68%
9	425(7.53%)	35(0.46%)	460	3.45%
10	322(5.70%)	45(0.59%)	367	2.76%
11	160(2.83%)	73(0.95%)	233	1.75%
12	1(0.02%)	23(0.30%)	24	0.18%
sum	5646	7670	13316	100.00%
