# Supplementary material for: Single-cell and spatial RNA sequencing reveal the spatiotemporal trajectories of fruit senescence
Source: Nat Commun. 2024 Apr 10;15:3108. doi: 10.1038/s41467-024-47329-x (PMC11006883; doi:10.1038/s41467-024-47329-x)
Supplement: Supplementary file 3 — Description of Additional Supplementary Files [file 41467_2024_47329_MOESM3_ESM.pdf]

Description of Additional Supplementary Files:

Supplementary Data 1: Cluster-enriched gene list and gene descriptions for 13 cell clusters.

Supplementary Data 2: Gene information identified in the spatial transcriptome profiles.

Supplementary Data 3: GO and KEGG of genes gathered in the cluster 3, 2 and 4 of pseudotime analysis.

Sheet 1 GO terms of genes gathered in cluster 3 of pseudotime analysis.

Sheet 2 KEGG terms of genes gathered in cluster 3 of pseudotime analysis.

Sheet 3 GO terms of genes gathered in cluster 2 of pseudotime analysis.

Sheet 4 KEGG terms of genes gathered in cluster 2 of pseudotime analysis.

Sheet 5 GO terms of genes gathered in cluster 4 of pseudotime analysis.

Sheet 6 KEGG terms of genes gathered in cluster 4 of pseudotime analysis.

Supplementary Data 4: Annotation and expression of genes in cluster 1 of the pseudotime analysis.

Supplementary Data 5: Annotation information for genes within 4 gene sets related to senescence and resistance.

Supplementary Data 6: KEGG analysis of genes in the branch related clusters of the pseudotime analysis.

Sheet 1 KEGG terms of genes in the cluster 1 of pseudotime analysis.

Sheet 2 KEGG terms of genes in the cluster 2 of pseudotime analysis.

Sheet 3 KEGG terms of genes in the cluster 3 of pseudotime analysis.

Sheet 4 KEGG terms of genes in the cluster 4 of pseudotime analysis.

Supplementary Data 7: Differentially expressed genes across senescence pseudotime.

Supplementary Data 8: GRN parameters of pseudotime related genes from Cytoscape.

Sheet 1 Parameters of nodes in the GRN.

Sheet 2 Clusters of GRN for pseudotime related genes by MCODE.

Sheet 3 Top20 nodes in the PRG network ranked by 12 methods of cytoHubba.

Sheet 4 Information on top10 nodes of GRN ranked by MNC.

Supplementary Data 9: Metabolic variations in different components in H. undatus analyzed by the SWATH method for mass spectrometry detection.

Sheet 1 Overview of metabolite information.

Sheet 2 Hierarchical classification information for all metabolites matched in the HMDB database.

Sheet 3 Information of top 30 metabolites of EX_vs_EN screened by VIP.

Sheet 4 Information of top 30 metabolites of ME_vs_EN screened by VIP.

Sheet 5 Information of top 30 metabolites of EX_vs_ME screened by VIP.

Supplementary Data 10: Expression of senescence related hub genes and primer sequences used in RTqPCR.

Sheet 1 Primer sequences used in RT-qPCR.

Sheet 2 Expression of senescence related hub genes in components of pericarp in the scRNA-seq profiles.
